# The influence factors on pit and fissure sealing behavior of 12-year-old children: a cross-sectional study in Zhejiang, China

**DOI:** 10.1186/s12887-024-04767-2

**Published:** 2024-08-02

**Authors:** Xin Ge, Huan Zhou, Xuejing Li, Lixuen Siow, Yanyi Xie, Yijie Hu, Yao Wan, Danli Fu, Haihua Zhu

**Affiliations:** 1 Stomatology Hospital, School of Stomatology, Zhejiang Provincial Clinical Research Center for Oral Diseases, Key Laboratory of Oral Biomedical Research of Zhejiang Province, Zhejiang University School of Medicine, Cancer Center of Zhejiang University, No.166 Qiutao North Road, Hangzhou, Zhejiang 310000 China; 2 Department of Prosthodontics, Shanghai Engineering Research Center of Tooth Restoration and Regeneration, Stomatological Hospital and Dental School of Tongji University, Shanghai, 200072 China

**Keywords:** Prevention of caries, Pit and fissure sealants, Preventive dentistry, Social determinants, Oral examination

## Abstract

**Background:**

In the 21st century, dental caries remains a global burden, particularly severely affecting the growth and quality of life of 12-year-old children. Fortunately, pit and fissure sealing (PFS) procedures can effectively prevent molars from caries. Hence, this study focused on the relationship between PFS and oral epidemiological factors in 12-year-old children.

**Methods:**

A cross-sectional survey was conducted in 12-year-old children from 11 cities in Zhejiang Province. Their dental conditions were collected through questionnaires, as well as basic information such as relevant family information, oral health knowledge and behavior. Then, logistic regression analysis was used to identify the influencing factors associated with PFS.

**Results:**

A total of 1204 children were included, with 252 in the PFS group and 952 in the non-PFS group. There were significant differences between the two groups in terms of decayed, missing and filled teeth (DMFT) score, first permanent molar DMFT score, residential area, educational level of parents, tooth-brushing frequency, use of dental floss, oral examination in a medical institution, having taken courses on oral health care, as well as having knowledge that tooth brushing could effectively prevent gingival inflammation, PFS could protect teeth, and oral disease may affect general health. According to further logistic regression analysis, the independent factors influencing PFS included use of dental floss [odds ratios (OR) = 1.672, 95% confidence intervals (CI) = 1.235–2.263, *P* = 0.001], having taken courses on oral health care (OR = 0.713, 95% CI = 0.515–0.988, *P* = 0.042), having knowledge that tooth brushing is effective in preventing gingival inflammation (OR = 0.627, 95% CI = 0.389–0.987, *P* = 0.044) and having knowledge that PFS can protect teeth (OR = 0.589, 95% CI = 0.438–0.791, *P* < 0.001).

**Conclusion:**

PFS can reduce the mean DMFT score of 12-year-old children. Independent influencing factors of PFS consist of use of dental floss, having taken courses on oral health care, oral health behavior and knowledge level.

## Background

As reported by the Global Burden of Disease Study 2016, dental caries in permanent teeth are characterized with the highest prevalence and second highest incidence of all diseases worldwide [1]. According to recommendation of the World Health Organization (WHO), 12 years old is considered as the target age for the detection of global caries [2]. It is worth noting that approximately 90% of caries in children’s permanent teeth occur in the pits and fissures of posterior teeth [3]. Insufficient mineralization, pits and fissures on the tooth surface can lead to accumulation of food residues and undisturbed biofilm, thereby increasing the risk of caries in children’s newly erupted teeth (particularly in the first permanent molars) within 2–4 years [4]. Luckily, pit and fissure sealing (PFS) procedures based on the National Oral Disease Intervention Plan for Children can effectively prevent molars from caries [5].

PFS have emerged as a potent intervention to prevent the onset and progression of dental caries in permanent teeth. These sealants work by forming a protective barrier over the occlusal surfaces of teeth, preventing food particles and bacterial by-products from accumulating in the pits and fissures, which are often sites of caries development. Ahovuo-Saloranta et al. conducted a systematic review and found strong evidence supporting the efficacy of PFS in reducing the incidence of caries in permanent molars in children and adolescents [5]. Similarly, Kashbour et al. compared the effectiveness of PFS with fluoride varnishes, another caries-preventive measure, concluding that PFS could significantly reduce caries incidence in the occlusal surfaces of permanent molars [6]. Emmanuelli et al. suggesed that a targeted approach in applying PFS, based on individual caries risk assessment, could enhance the efficiency of PFS as a preventive tool [7]. The introduction of PFS into preventive dentistry has revolutionized the approach toward managing the risk of dental caries in permanent teeth, especially in high-risk populations such as children and adolescents. The evidence base supporting the efficacy and cost-effectiveness of PFS reinforces the importance of incorporating this intervention into comprehensive oral health programs.

In this study, we not only investigated the relationship between PFS rates and oral epidemiological factors in 12-year-old children but also discussed other aspects such as the single-child family and left-behind children. This study provided supports for the implementation of targeted measures to promote the oral health of children.

## Materials & methods

### Ethical considerations

The Oral Health Survey scheme in Zhejiang Province was approved by the Stomatological Ethics Committee of the Chinese Stomatological Association and the Ethics Committee of the Stomatology Hospital Affiliated to Zhejiang University School of Medicine (No. 2020-37). All subjects were involved in this scheme voluntarily. An information sheet was circulated to the families and/or legal guardians of the study participants, followed by the acquirement of their informed written consent.

### Study sample size

A cross-sectional survey was employed to investigate the correlation of PFS rates with oral health behavior, sociodemographic characteristics, oral health knowledge, and attitudes of 12-year-old children.

The required sample size was calculated according to the formula as follows:

$${\rm{n}} = deff\frac{{\mu a2p(1 - p)}}{{{\delta ^2}}}$$

In this formula, n represented the sample size, *deff* was the design effect set at 4.5, *p* was the dental caries prevalence set as 40.9% (according to the Fourth National Oral Survey of Zhejiang Pronvince), *µ* was the level of confidence (1.96), and *δ* was the margin of error. The non-response rate was set at 20.0%. Based on this estimation, the final sample size required was 1,200.

### Participants

Probability proportional sampling (PPS) is a multi-stage, stratified, random, and quota sampling method. In this study, the participants were randomly selected from 11 cities in Zhejiang Province in terms of their population size using the PPS method. The inclusion criteria of participants were shown as follows: patients (1) giving informed consent for the oral examination and filling out questionnaire; (2) having ability to cooperate in the oral examination without vomiting, coughing, etc.; (3) aged 12 years old. The participants were divided into the PFS group and non-PFS group depending on whether PFS had been performed.

### Oral examination

A clinical assessment for the oral conditions of children was performed by dentists according to the methods and standards provided by the WHO Oral Health Survey guidelines. The basic information recorded included name, gender, date of birth, ID number, and nationality. The oral examination was conducted by three trained and calibrated dentists, two of whom were responsible for recording the examination results. Briefly, the subjects were in a supine position in a mobile dental chair in a school classroom, and the examination was performed using a mobile light source, a disposable flat oral mirror, and a community periodontal index (CPI) probe.

To evaluate the oral conditions, decayed teeth (DT), missing teeth (MT), filled teeth (FT), and decayed, missing and filled teeth (DMFT) were examined by dentists. After examination, permanent teeth injury, PFS, gingival bleeding, and dental calculus were scored as “1 = Yes” or “0 = No.” The gingival bleeding and probing calculus rates were used to assess periodontal health, and the percentage of children with DMFT ≥ 1 score was considered as the prevalence rate of dental caries. The participants were told with their oral conditions by dentists before they filled out a structured questionnaire.

### Concurrent questionnaire survey

All participants were asked to fill out a structured questionnaire after the oral examination. The questionnaire included the information as follows: the relevant family information (single-child family, left-behind children, and the education level of parents); oral health behaviors and diet habits (frequency of brushing teeth, use of fluoride toothpaste, use of dental floss, and frequency of eating sweets); caries lesions (whether had dental caries), medical experience, and self-assessment of oral condition (whether there was a tooth injury or toothache, dental treatment experience, and self-assessment of oral condition as average/healthy/poor); and knowledge and attitude related to oral health. The oral knowledge survey included eight questions, all of which were answered as “yes” or “no”: (a) “Is it normal for gums to bleed when brushing teeth?”, (b) “Is gum infection caused by bacteria?”, (c) “Is brushing useless for preventing gum infection?”, (d) “Are dental caries caused by bacteria?” (e) “Are cavities caused by sugar?”, (f) “Is fluoride ineffective for tooth protection?”, (g) “Can pit and fissure sealants protect teeth?”, and (h) “Do oral diseases affect overall health?”. Four statements were used to evaluate the importance of oral health in the quality of life, the necessity of regular dental examinations, the heritability of tooth quality, and the importance of personal efforts in dental protection. The scores for dental knowledge and attitude were determined as the sum of correct answers. Participants were also asked to answer the question: “Did you take any oral healthcare courses last semester?”

### Quality control

Before the investigation, all three examiners received the same theoretical and clinical knowledge training. Each examiner was then calibrated against the standard examiner and another examiner by evaluating 20 children, until the kappa value used to determine inter-examiner reproducibility exceeded 0.85. Additionally, in an oral survey of each school, 5% of the samples were randomly re-examined, and kappa values were recorded to monitor the reproducibility among examiners.

### Statistical analysis

SPSS statistical software (version 24.0; IBM Corporation, Armonk, NY) was used for the statistical analysis of all data. The children with pit and fissure were labeled as PFS group and the children without pit and fissure were labeled as non-PFS group. A chi-square analysis was conducted to evaluate the differences between the PFS group and non-PFS group in terms of caries prevalence, gingival bleeding rate, and calculus rate. Since these caries scores did not show a normal distribution, the Mann–Whitney U test or Kruskal–Wallis one-way analysis of variance was used to compare the average DMFT scores between the two groups. Logistic regression analysis was performed to explore the correlation of the PFS rate with oral status, oral health attitudes and knowledge. The analysis results were expressed as odds ratios (OR) and 95% confidence intervals (CI). *P* < 0.05 indicated a statistical significance.

## Results

### The oral health status assessments in PFS group and non-PFS group

A total of 1361 questionnaires were collected, among which 1204 questionnaires were complete and correct. Of them, there were 252 questionnaires in the PFS group and 952 in the non-PFS group, with a survey response rate of 88.5%. The reliability of oral health status assessments, as measured by kappa statistics, ranged from 0.84 to 0.93. The overall prevalence of dental caries was 45.1%. The prevalence of oral diseases and dental caries scores were evaluated in the PFS group and non-PFS group. The results showed that the overall caries rate was similar between 12-year-old children with and without PFS (*P* = 0.603). Moreover, the caries rate in the first permanent molars was not significantly different between the two groups (*P* = 0.828). Besides, compared with the non-PFS group, the PFS group had a lower mean DMFT score (1.004 ± 1.479 vs. 1.289 ± 2.053, *P* = 0.039) and a lower mean DMFT score in the first permanent molar (0.623 ± 0.934 vs. 0.787 ± 1.185, *P* = 0.042) (Table 1).

**Table 1:** The relationship between PFS and tooth conditions

	PFS (*n* = 252)	Non-PFS (*n* = 952)	t/Chi-square value	*P*-value
DMFT ≥ 1	110 (43.7%)	433 (45.4%)	0.270	0.603
DMFT score (‾x ± SD)	1.004 ± 1.479	1.289 ± 2.053	2.066	**0.039**
DMFT ≥ 1 (First permanent molar)	95 (37.7%)	366 (38.4%)	0.047	0.828
DMFT score (First permanent molar ‾x ± SD)	0.623 ± 0.934	0.787 ± 1.185	2.036	**0.042**

Abbreviations: DMFT: decayed, missing, and filled teeth; PFS: pit and fissure sealing

### The association of pit and fissure sealing rate with oral health behavior and sociodemographic characteristics

The correlation of the PFS rate with oral health behavior and sociodemographic characteristics was shown in Table 2. PFS was statistically significantly associated with 12-year-old children’s residential areas, parents’ education level, brushing frequency, use of dental floss, oral examinations at a medical institution, and a experience of taking on oral health care courses (all *P* < 0.01). Children from cities, whose fathers and mothers both received higher education, who brushed their teeth twice or more times per day, who used dental floss, and who visited medical institutions for oral examinations had higher sealed rates of pit and fissures. PFS had no significant association with gender, single-child family, left-behind children, the brushing methods used, use of fluoride toothpaste, the frequency of eating sweets, dental pain, or dental injured (all *P* > 0.05).

**Table 2:** The correlation of pit and fissure sealing rate with oral health behavior and sociodemographic characteristics

Variable	Total (*n* = 1204)	PFS (*n* = 252)	Non-PFS (*n* = 952)	Chi-square value	*P*
**Gender**				0.022	0.882
Boy	607 (50.4%)	126 (50%)	481(50.5%)		
Girl	597 (49.6%)	126 (50%)	471(49.5%)		
**Single-child family**				0.715	0.398
Yes	389 (32.3%)	87 (34.5%)	302(31.7%)		
No	815 (67.7%)	165 (65.5%)	650(68.3%)		
**Residential area**				20.634	**< 0.001**
Urban	812 (67.4%)	200 (79.4%)	612(64.3%)		
Rural	392 (32.6%)	52 (20.6%)	340(35.7%)		
**Paternal educational level**				11.806	**0.001**
Junior college degree or below	979 (81.3%)	186 (73.8%)	793(83.3%)		
Bachelor degree or above	225 (18.7%)	66 (26.2%)	159(16.7%)		
**Maternal education level**				15.124	**< 0.001**
Junior college degree or below	981 (81.5%)	184(73.0%)	797(83.7%)		
Bachelor degree or above	223 (18.5%)	68 (27.0%)	155(16.3%)		
**Parents work in other places**				0.095	0.758
At least one parent works in another places	339 (28.2%)	69 (27.4%)	270(28.4%)		
Neither parent works in other places	865 (71.8%)	183 (72.6%)	682(71.6%)		
**Tooth-brushing duration**				1.980	0.159
Brushing for 3 min or more	314 (26.1%)	57 (22.6%)	257(27.0%)		
Brushing for less than 3 min	890 (73.9%)	195 (77.4%)	695(73.0%)		
**Tooth-brushing method**				0.919	0.338
Correct brushing methods: Circle method, Modified bass brushing technique	320 (26.6%)	61 (24.2%)	259(27.2%)		
Incorrect brushing: horizontal brushing	884 (73.4%)	191 (75.8%)	693(72.8%)		
**Tooth-brushing frequency**				7.768	**0.005**
≥ Twice per day	642 (53.3%)	154 (61.1%)	488(51.3%)		
≤ Once per day	562 (46.7%)	98 (38.9%)	464(48.7%)		
**Use dental floss**				24.615	**< 0.001**
Yes	354 (29.4%)	106 (42.1%)	248(26.1%)		
No	850 (70.6%)	146 (57.9%)	704(73.9%)		
**Use fluoride toothpaste**				3.392	0.066
Yes	224 (18.6%)	57 (22.6%)	167(17.5%)		
No	980 (81.4%)	195 (77.4%)	785(82.5%)		
**Frequency of eating** **desserts and candies**				0.036	0.850
At least once a day	286 (23.8%)	61 (24.2%)	225(23.6%)		
Less than once a day	918 (76.2%)	191 (75.8%)	727(76.4%)		
**Frequency of consuming sweetened drinks**				0.301	0.83
At least once a day	118 (9.8%)	27 (10.7%)	91(36.1%)		
Less than once a day	1086(90.2%)	225 (89.3%)	861(63.9%)		
**Frequency of consuming sweetened milk and yogurt**				0.562	0.454
At least once a day	224 (18.6%)	51 (20.2%)	173(18.2%)		
Less than once a day	980 (81.4%)	201 (79.8%)	779(81.8%)		
**Toothache experience**				0.348	0.555
Yes	598 (49.7%)	121 (48.0%)	477(50.1%)		
No	606 (50.3%)	131 (52.0%)	475(49.9%)		
**Tooth injury**				0.596	0.440
Yes	196 (16.3%)	37 (14.7%)	159(16.7%)		
No	1008(83.7%)	215 (85.3%)	793(83.3%)		
**Oral examination in a medical institution**				20.048	**< 0.001**
Yes	809 (67.2%)	199 (79.0%)	610(64.1%)		
Never	395 (32.8%)	53 (21.0%)	342(35.9%)		
**Had taken courses on oral health care in the past year**				8.737	**0.003**
Yes	279 (23.2%)	76 (30.2%)	203(21.3%)		
No	925 (76.8%)	176 (69.8%)	749(79.7%)		

### The relevance of pit and fissure sealing rate to oral health knowledge, attitude, and self-assessment of oral conditions

As shown in Table 3, a questionnaire survey was adopted to analyze the correlation of the PFS rate with oral health knowledge, attitude, and self-assessment of oral conditions. In brief, a higher PFS rate was associated with correct answers to questions on the effectiveness of brushing in preventing gingival inflammation (*P* = 0.014), whether PFS could protect teeth (*P* < 0.001), and whether oral disease affected general health (*P* < 0.001).

**Table 3:** The relationship between pit and fissure sealing rate and oral health knowledge, attitude, and self-assessment of oral conditions

Variables	Total (*n* = 1204)	PFS (*n* = 252)	Non-PFS (*n* = 952)	Chi-square value	*P*
Oral health knowledge					
**1. Gum bleeding is normal when brushing teeth**				1.211	0.271
Correct answer	733 (60.9%)	161 (63.9%)	572(60.1%)		
Wrong answer	471 (39.1%)	91 (36.1%)	380(39.9%)		
**2. Bacteria can cause gingivitis**				3.621	0.057
Correct answer	998 (82.9%)	219 (86.9%)	779(81.8%)		
Wrong answer	206 (17.1%)	33 (13.1%)	173(18.2%)		
**3. Tooth brushing is effective in preventing gingival inflammation**				6.068	**0.014**
Correct answer	1020 (84.7%)	226 (89.7%)	794(83.4%)		
Wrong answer	184 (15.3%)	26 (10.3%)	158(16.6%)		
**4. Bacteria can cause dental caries**				3.108	0.078
Correct answer	804 (66.8%)	180 (71.4%)	624(65.5%)		
Wrong answer	400 (33.2%)	72 (28.6%)	328(34.5%)		
**5. Eating sugar can cause dental caries**				0.001	0.981
Correct answer	918 (76.2%)	192 (76.2%)	726(76.3%)		
Wrong answer	286 (23.8%)	60 (23.8%)	226(23.7%)		
**6. Fluoride does not protect teeth**				2.835	0.092
Correct answer	694 (57.6%)	157(62.3%)	537(56.4%)		
Wrong answer	510 (42.4%)	95(37.7%)	415(43.6%)		
**7. Pit and fissure sealing can protect teeth**				27.207	**< 0.001**
Correct answer	514 (42.7%)	144 (57.1%)	370(38.9%)		
Wrong answer	690 (57.3%)	108 (42.9%)	582(61.1%)		
**8.Oral disease may affect general health**				12.991	**< 0.001**
Correct answer	876 (72.8%)	206 (81.7%)	670(70.4%)		
Wrong answer	328(27.2%)	46(18.3%)	282(29.6%)		
Oral health attitudes					
**1. Oral health is important to your life**				0.310	0.578
Correct answer	1185 (98.4%)	249 (98.8%)	936(98.3%)		
Wrong answer	19 (1.6%)	3 (1.2%)	16(1.7%)		
**2. Regular oral examination is very important**				1.183	0.227
Correct answer	1092(90.7%)	233 (92.5%)	859(90.2%)		
Wrong answer	112 (9.3%)	19 (7.5%)	93(9.8%)		
**3. Tooth quality is innate and has nothing to do with taking measures to prevent caries**				0.250	0.617
Correct answer	1123 (93.3%)	237 (94.0%)	886(93.1%)		
Wrong answer	81 (6.7%)	15 (60.%)	66(6.9%)		
**4. Prevention of dental disease depends on oneself first**				0.003	0.957
Correct answer	1157 (96.1%)	242 (96.0%)	915(96.1%)		
Wrong answer	47 (3.9%)	10 (4.0%)	37(73.9%)		
**Self-oral evaluation**				5.115	0.077
Very good/Good	378 (31.4%)	88 (34.9%)	290(30.5%)		
Average	635 (52.7%)	135 (53.6%)	500(52.5%)		
Poor/Very poor	191 (15.9%)	29 (11.5%)	162(17.0%)		

### Logistic regression analysis of the influencing factors related to pit and fissure sealing

The above-mentioned indicators with significant differences were subject to the logistic regression analysis to screen factors influencing PFS. As displayed in Table 4, the main factors influencing PFS included use of dental floss (OR = 1.672, 95% CI = 1.235–2.263, *P* = 0.001), having taken courses on oral health care (OR = 0.713, 95% CI = 0.515–0.988, *P* = 0.042), as well as having knowledge that tooth brushing is effective in preventing gingival inflammation (OR = 0.627, 95% CI = 0.389–0.987, *P* = 0.044) and PFS can protect teeth (OR = 0.589, 95% CI = 0.438–0.791, *P* < 0.001).

**Table 4:** Logistic regression analysis of the risk factors related to pit and fissure sealing

Variables	B	SE	Wald statistic	*P*	OR	95% CI	
						Lower	Upper
Oral examination in a medical institution? (Yes)	0.485	1.264	0.147	0.701	1.625	0.136	19.364
Urban vs. Rural (Urban)	-0.956	1.272	0.565	0.452	0,384	0.032	4.649
Paternal educational level (Junior college degree or below)	0.148	0.233	0.402	0.526	1.159	0.734	1.832
Maternal education level (Junior college degree or below)	0.455	0.232	3.854	0.050	1.577	1.001	2.485
Tooth-brushing frequency(≥ Twice per day)	-0.167	0.152	1.213	0.271	0.846	0.628	1.139
Use dental floss (Yes)	0.514	0.155	11.055	**0.001**	1.672	1.235	2.263
Had taken courses on oral health care in the past year	-0.338	0.166	4.138	**0.042**	0.713	0.515	0.988
Tooth brushing is effective in preventing gingival inflammation (Correct answer)	-0467	0.232	4.059	**0.044**	0.627	0.389	0.987
Does pit and fissure sealing protect teeth? (Yes)	-0.530	0.150	12.404	**< 0.001**	0.589	0.438	0.791

Abbreviations: B, regression coefficient; P, significance level; OR, odds ratio; CI, confidence interval

## Discussion

Maintaining good oral health is crucial for the appropriate growth and development of 12-year-old children who have early permanent teeth [8]. In previous studies, application of sealants reduced the incidence of caries after 2 years or more of follow-up, which proved the effectiveness and safety of sealants in preventing or stopping the progression of non-vacuolar caries, as compared to controls without sealants [9, 10]. In this cross-sectional study, we investigated the relevance of PFS rates to oral epidemiological factors in 12-year-old children in Zhejiang Province, China. Specifically, the overall prevalence of dental caries was 45.1%; the PFS group exhibited a lower mean DMFT score (1.004 ± 1.479 vs. 1.289 ± 2.053) and a lower mean DMFT score in the first permanent molar (0.623 ± 0.934 vs. 0.787 ± 1.185) than the non-PFS group. Such outcomes indicated that PFS procedures could help reduce the DMFT score. Further logistic regression analysis revealed that use of dental floss, having taken courses on oral health care, oral health behavior and knowledge level were independent influence factors associated with PFS.

The results of this survey showed that the proportion of PFS in children living in urban areas was higher than that in children living in rural areas (79.4% vs. 20.6%). Besides, children whose parents had a higher education level (bachelor’s degree or above) also had higher proportions of PFS (26.2%). Notably, urban parents with greater educational attainment are more likely to focus on their children’s oral health and have a better cognition of oral health behavior and knowledge of PFS [11]. This highlights the significance of enhancing the scope and intensity of oral education, the form of education, as well as the public’s oral health attitudes and knowledge levels. However, there was no discernible difference in the use of PFS among children with or without siblings. With the development of economy, parents often work in cities far from home, and the physical and mental health of left-behind children needs to be concerned. In this paper, we found that the proportion of PFS among children with parents who worked in their resided cities was slightly higher than that among children with parents who worked in other cities (21.1% vs. 20.4%) [12].

Children’s oral health knowledge and behavior have been shown to be important factors in promoting PFS [13]. For example, children who brushed their teeth twice or more a day had a higher proportion of PFS than those who brushed their teeth once a day or less (24.0% vs. 17.4%). The proportion of children who used dental floss also had a higher proportion of PFS than that of those who did not (29.9% vs. 17.2%); children who had an oral examination in a medical institution were more likely to receive PFS than those who never had (24.6% vs. 13.4%). Overall, children who more frequently underwent PFS exhibited better oral health behaviors. Among the 12-year-old children in our study, those who believed that brushing could prevent gingival inflammation (26.1% vs. 14.1%), PFS could protect teeth (28.0% vs. 15.7%), and oral health could affect general health (23.5% vs. 14.0%) had a higher proportion of PFS than those who did not. Further logistic regression analysis revealed that use of dental floss, having taken courses on oral health care, oral health behavior and knowledge level were independent influencing factors associated with PFS.

As is known to all, use of dental floss, PFS behavior and oral health behavior are associated with oral health knowledge and a experience of having oral health education courses. Oral diseases have a strong social and behavioral profile, highlighting the significance of implementing educational interventions that encourage health behaviors to promote the prevention of oral diseases [14, 15]. Finnegan et al. reported that the oral health knowledge of caregivers had an influence on the risk of dental caries development in children [16]. A research in the United States demonstrated that regardless of socioeconomic status, grandparent caregiver’s oral health knowledge could positively affect their oral health-related behaviors and values, thereby influencing their grandchildren’s’ oral health [17]. Zhao et al.’s study revealed a relevance between children’s oral health knowledge and good oral health-related quality of life, in which children’s oral health self-efficacy and behaviour had indirect effects [18]. Healthy lifestyle habits, such as daily brushing, regular exposure to sources of fluoride and moderate intake of sugar, are the most effective ways to prevent caries lesions. Therefore, it is necessary to expand children’s oral health knowledge through a variety of avenues, such as school, family, and media, thereby improving their oral health behavior.

The efficacy of PFS in preventing dental decay in permanent teeth, especially in children and adolescents, has been extensively documented. Ahovuo-Saloranta et al. provided a comprehensive systematic review, affirming that PFS significantly reduce caries incidence in the occlusal surfaces of molars, a common site for caries development due to their anatomical complexity [5]. Kashbour et al. concluded that both PFS and fluoride varnishes are effective in caries prevention, though their comparative effectiveness may vary depending on the tooth surface and individual’s caries risk [6]. Rashed et al. echoed these findings, suggesting that while fluoride varnish is beneficial for smooth surfaces, PFS offers superior protection for occlusal surfaces [19]. Hesse et al. conducted a randomized clinical trial that demonstrated the effectiveness of sealing over partial caries removal in primary molars [20]. This study not only supports the use of PFS as a preventive measure but also challenges traditional caries management strategies, advocating for a more conservative approach that prioritizes the preservation of tooth structure. However, from this study, we can see that the popularity of PFS is insufficient. In China, PFS is implemented by two types of organizations: hospitals and schools [21]. A school-based PFS plan is an effective intervention for improving children’s oral health and a health-promotion strategy for enhancing oral health in general. Such plan is also applicable to other developing countries where the best intervention measures are not universally applied [22]. Generally, the developing countries have a high caries rate due to insufficient primary prevention publicity related to oral diseases [23]. The school-based prevention programs are worthwhile in developing countries where social and economic burden of oral disease is particularly high [24].

## Conclusion

Overall, PFS can reduce the mean DMFT score among 12-year-old children. Besides, independent influencing factors associated with PFS consists of use of dental floss, having taken course on oral health care, oral health behavior and knowledge level. Our research supports the continued implementation of policies to improve the oral health of children. Therefore, it is necessary to increase financial support for school-based oral healthcare and implementation of comprehensive oral intervention programs for school-age children, as well as to establish a national school oral health service network.

## Data Availability

The datasets used and analysed during the current study are available from the corresponding author on reasonable request.

## Declarations

All methods were performed in accordance with relevant guidelines and regulations. All the participation was voluntary. An information sheet was circulated to and informed consent for their data to be used for research purposes. Informed written consents was obtained from all families or legal guardians of children participating in the study.

## Abbreviations

CI: Confidence interval
OR: Odds ratio
PFS: Pit and fissure sealing
SD: Standard deviation
DMFT: Decayed, missing, and filled teeth
DT: Decayed teeth
MT: Missing teeth
FT: Filled teeth
